# Parsimony and Poeciliid Sex Chromosome Evolution

**DOI:** 10.1093/gbe/evad128

**Published:** 2023-09-06

**Authors:** Lydia J M Fong, Iulia Darolti, David C H Metzger, Jake Morris, Yuying Lin, Benjamin A Sandkam, Judith E Mank

**Affiliations:** Department of Zoology and Biodiversity Research Centre, University of British Columbia, Vancouver, Canada; Department of Ecology and Evolution, University of Lausanne, Switzerland; Department of Zoology and Biodiversity Research Centre, University of British Columbia, Vancouver, Canada; School of Biological Sciences, University of Bristol, United Kingdom; Department of Zoology and Biodiversity Research Centre, University of British Columbia, Vancouver, Canada; Department of Neurobiology and Behavior, Cornell University, Ithaca, New York, USA; Department of Zoology and Biodiversity Research Centre, University of British Columbia, Vancouver, Canada

Occam's Razor is a philosophical guide that recommends that the simplest possible explanation should be concluded when confronted with multiple plausible scenarios. In Evolutionary Biology, Occam's Razor is also known as the Principle of Parsimony or the Law of Parsimony, and it underpins many aspects of the field, from ancestral state reconstruction across phylogenies to model fitting. Importantly, Occam's Razor leaves the door open to new data coming to light in the future supporting alternative models.

**Fig. 1.**
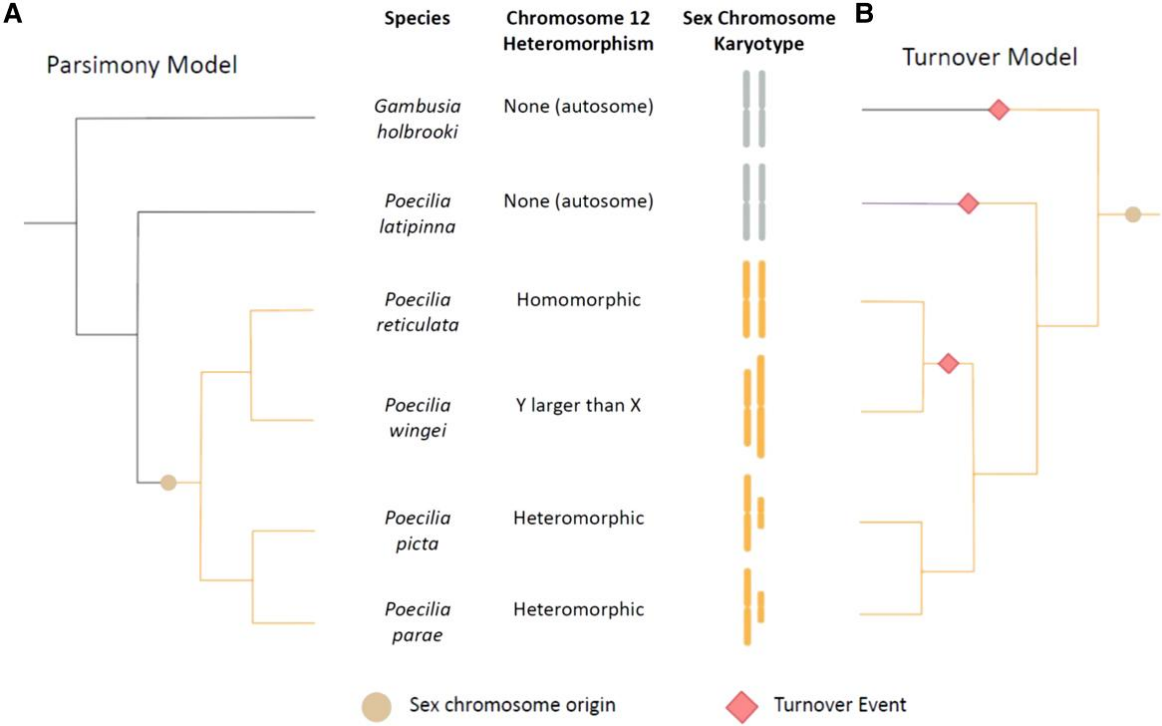
Illustration of two proposed models for the evolution of sex chromosomes in a clade of Poeciliid fishes, parsimony (*A*) and turnover (*B*). The circle represents the proposed sex chromosome origin and red diamonds represent some of the necessary turnover events for this model. Colored lines indicate the linkage group containing the sex chromosome, with orange for guppy chromosome 12 and grey for all other chromosomes.

Occam's Razor is relevant to the Poeciliid sex chromosomes because several models have been proposed to explain their remarkable diversity in Y chromosome degeneration (Darolti et al. 2019; Charlesworth, Bergero, et al. 2021; Kirkpatrick et al. 2021; Metzger et al. 2021). Several species have sex chromosomes on the same linkage group, with some retaining largely homomorphic sex chromosomes, with others exhibiting extensive heteromorphism (fig. 1). The simplest explanation for this diversity, in other words the one requiring the fewest evolutionary steps, is a single recent origin in the immediate ancestor of the group, roughly 20 million years ago. Notably, Charlesworth, Bergero, et al. (2021) have argued instead for an ancient origin with several subsequent turnover events. Sex chromosomes have been shown to evolve via many different routes (Furman et al. 2020), and fish in particular show great diversity in sex determination (Mank et al. 2006). These alternative models, and others, are therefore possible, and all are testable with available data.

An examination of the available data shows consistent support for a single recent origin. First, maximum parsimony reconstructions based on the presence of degenerate sex chromosomes all point to a single recent origin (Darolti et al. 2019; Metzger et al. 2021). Importantly, all the sex chromosomes assessed within the clade share the same pseudoautosomal region (PAR) boundaries (Darolti et al. 2019), suggesting that recombination ceased just proximal to that in the ancestor and has progressed at different rates in daughter lineages.

Additionally, an ancient origin of recombination suppression in a distant ancestor of *Poecilia picta* would necessitate many more turnover events than just in *Poecilia reticulata*. Although Charlesworth, Bergero, et al. (2021) have not suggested exactly when recombination suppression and Y chromosome degeneration might have occurred, they have previously indicated that it predates the split of *Poecilia* and *Xiphophorus* (Bergero et al. 2019). However, none of the outgroups nearest to the *P. reticulata–P. picta* clade has degenerated sex chromosomes or even share the same sex chromosome system (Darolti et al. 2019). Notably, none of the *Xiphophorus* species thus far examined share the guppy sex chromosome location. All this necessitates many more turnovers than shown in figure 1. In fact, of all Poeciliids examined thus far, this linkage group is only degenerated in *P. picta* and its closest relatives.

Furthermore, if recombination suppression occurred in a distant ancestor, followed by turnover events, we might expect the ancestral X to retain molecular signatures of being a sex chromosome (Vicoso and Bachtrog 2013) that are evident on the *P. picta* X. However, outgroup lineages lack molecular signatures of degenerate sex chromosomes observed in *P. picta* (Metzger et al. 2022; Darolti et al. 2023), again only consistent with a recent origin.

We further tested the possibility of an ancient origin of the *P. picta* sex chromosomes followed by turnover events in Fong et al. (2023). We first estimated synonymous substitution (*d_S_*) rate for sex-linked orthologs found between *P. reticulata* and *P. picta*. *d_S_* has been successfully used as a relative measure of time since recombination suppression in a wide variety of species (Lahn and Page 1999; Bergero et al. 2007, Wright et al. 2014). If there was an ancient origin to the *P. picta* sex chromosome, we would expect nonoverlapping and significantly higher *d_S_* values between the X–Y orthologs found in *P. picta* relative to *P. reticulata*. However, the *P. picta* X–Y *d_S_* values were not statistically different than those in *P. reticulata* or *Poecilia wingei* (Darolti et al. 2020), suggesting recombination suppression occurred at similar times on their sex chromosomes.

Although Charlesworth et al. (2023) do concede that the divergence values we estimate support the conclusion of recent recombination suppression, they raise some methodological concerns. Even minor details of methods have proved critical in detecting patterns of sex chromosome divergence in guppies (Darolti et al. 2022), and so it is important to understand every feature.

First, Charlesworth et al. (2023) suggest that the X–Y orthologs we identified may be located within the PAR that still recombines and has not degenerated. Had we, as Charlesworth et al. (2023) assume, mapped these genes to the *P. reticulata* genome or a *P. picta* genome that had been scaffolded with the *P. reticulata* genome (Charlesworth, Graham, et al. 2021; Künstner et al. 2016), we might indeed expect the spurious inclusion of PAR genes in the nonrecombining region. However, our approach avoided this potential technical artifact by mapping the X–Y orthologs to our newly produced chromosome-level female *P. picta* reference genome that was *de novo* assembled from PacBio HiFi reads scaffolded with Hi-C data, thus ensuring we captured any inversions unique to *P. picta* (Metzger et al. 2022). We used this new *P. picta* reference genome for detailed synteny analysis with related species, including *P. reticulata*, to ensure an accurate PAR boundary. More importantly, we were also careful to delineate the PAR as the region without Y degeneration based on read depth differences between males and females. Using read depth differences, we estimated the PAR boundary at ∼30 Mb (Fong et al. 2023, supplemental figure 2) and removed all genes from this region for further analysis. Based on M:F read coverage, the Y chromosome in the 25- to 30-Mb region adjcent to the PAR and syntenic with the *P. reticulata* non-recombining region is completely degenerated, and this region is not a PAR, as stated in the Materials and Methods of Fong et al. (2023). Most importantly, X–Y *d_S_* estimates in this region are significantly greater than 0 and therefore must be in the nonrecombining region of *P. picta* as well.

We also used shared male-specific k-mers to test alternative models of Y evolution. Male-specific k-mers, often referred to as Y-mers, can reveal much about the male-specific region of the Y chromosome (Caravalho and Clark 2013). Y-mers shared among multiple species suggest shared ancestry of the Y, and this approach has been used in a range of species (Morris et al. 2018; Torres et al. 2018; Sandkam et al. 2021; Kabir et al. 2022). We would not expect shared Y-mers between *P. picta* and *P. reticulata* if the Y chromosome of *P. reticulata* represented a turnover event. However, we detect a pattern of shared Y-mers only consistent with shared ancestry of the Y.

Charlesworth et al. (2023) suggest that “considerable enrichment of repeats in the completely Y-linked region, readily accounting for a small number of male-specific k-mers” and “after genes had lost functions, which would also be likely to delete repetitive sequences, reducing number of male-specific k-mers.” This is not true and conflates k-mer coverage approaches (where k-mers are present in both male and female genomes, but in different abundances, which we addressed in Fong et al. 2023, supplemental figure 4) with male-specific k-mers, or Y-mers (Fong et al. 2023, figure 4). In fact, the number of Y-mers (either within or shared across species) does not reflect the size of the Y as these approaches exclude many true Y-linked k-mers if they happen to match the sequence of autosomal- or X-linked k-mers. Thus, rather than indicating Y size, Y-mer numbers indicate the amount of male-unique sequence that is distinct from the X and autosomes.

Charlesworth et al. (2023) suggest that repeat elements enriched on a Y chromosome would elevate the number of Y-mers, but this is only true if the repeat sequence is unique to the Y chromosome and absent from all other areas of the genome. Even one copy of a repetitive element on the X or autosomes would remove the sequence from the Y-mer catalog. More importantly, these elements would not produce Y-mers that are shared across species unless those repetitive elements diverged in a common ancestor, inconsistent with turnover of the Y chromosome. Therefore, the pattern of shared Y-mers presented in figure 4 of Fong et al. (2023) is only consistent with shared ancestry of the Y chromosome.

Charlesworth et al. (2023) suggest a theoretical timeline of degeneration that would be expected for a Y chromosome based on a gene number model (Bachtrog 2008) and propose this predicts an older origin of the sex chromosome. First, it is worth noting that such a model relies on knowing the number of genes on the Y. As we do not yet have a complete Y chromosome gene catalog, we do not know how many genes have been silenced, deleted, or otherwise lost from the *P. picta* Y chromosome. Moreover, with reference to the gene number model, Bachtrog (2008) states that “only a very simple scenario of Y evolution is explored and I ignore evolutionary responses on the X chromosome, such as the acquisition of dosage compensation.” This is highly relevant to the Poeciliid sex chromosome because dosage compensation has recently been shown to be capable of fostering rapid rates of degeneration (Lenormand and Roze 2022). Even more importantly, the species in the clade with highly degenerate Y chromosomes also all exhibit complete X chromosome dosage compensation (Darolti et al. 2019; Metzger et al. 2021), and an enrichment on the X chromosome of a transposon carrying a dosage-compensation motif (Metzger et al 2022). Although Charlesworth et al. (2023) rightly point out that not all aspects of how dosage compensation might accelerate Y degeneration have been modeled, the fact remains that theoretical models predict that dosage compensation can promote rapid Y degeneration, providing a plausible explanation for the variation observed in Poeciliids.

Finally, Charlesworth et al. (2023) suggest from their title that the 20 million years since the origin of the Poeciliid sex chromosomes represents “an evolutionary instant” that is too short to permit the rapid degeneration of a sex chromosome from a recent single origin. However, we note recent work has shown similar rapid expansion of the nonrecombining region on the Y chromosome shared by Cannabis and Hemp in just 12–28 million years (Prentout et al. 2021), again coupled with the evolution of dosage compensation. This suggests a broader pattern spanning diverse taxa of sex chromosome divergence in the context of dosage compensation.

In conclusion, although we fully agree that further data may support alternative explanations, in the absence of such data, a single recent origin of the sex chromosomes remains the simplest (i.e., most parsimonious) explanation of all the available data, and this model is consistent with recent theoretical work and recent evidence from other species. We thank Charlesworth et al. for their thought-provoking engagement in the discussion about potential models of sex chromosome evolution in the Poeciliids and look forward to future data with which alternative models can be further evaluated.
